# Species diversity and competition influence nitrogen resorption efficiency in mixed hardwood plantations

**DOI:** 10.1007/s00442-026-05873-x

**Published:** 2026-03-14

**Authors:** Taylor M. Nelson, Minjee Park, Lorenzo Cotrozzi, Kliffi M. S. Blackstone, Gordon G. McNickle, Douglass F. Jacobs, John J. Couture

**Affiliations:** 1 Department of Entomology, Purdue University, West Lafayette, IN USA; 2 Department of Forestry and Natural Resources, Purdue University, West Lafayette, IN USA; 3 Center for Plant Biology, Purdue University, West Lafayette, IN USA; 4 Department of Botany and Plant Pathology, Purdue University, West Lafayette, IN USA; 5 Department of Agriculture, Food and Environment, Universitá Di Pisa, Pisa, Italy

**Keywords:** Diversity, Forest nutrient dynamics, Interspecific competition, Intraspecific competition, Nitrogen resorption efficiency

## Abstract

Nutrient dynamics substantially influences forest productivity, yet the influence of competition on these processes in forests is not well understood. Nitrogen is an important limiting nutrient for tree growth, but how canopy nitrogen dynamics are influenced by tree diversity and competition is unclear. Nitrogen-use efficiency is one mechanism suggested to support niche complementarity, promoting increased productivity as diversity increases, and is influenced by the resorption of foliar nutrients during senescence. We tested how diversity and competition affect canopy nitrogen dynamics by examining nitrogen resorption efficiency (NRE) in over 200 trees from three tree species in a 12-year-old mixed-species hardwood planting that included a full-factorial design of three levels of species diversity, representing intra- and interspecific competition, and two levels of planting density, representing competition intensity. Midseason and senescent foliar samples were collected from black cherry (*Prunus serotina*), American chestnut (*Castanea dentata*), and northern red oak (*Quercus rubra*) and we determined midseason canopy and foliar litter nitrogen concentrations and calculated NRE. Higher midseason canopy nitrogen concentrations were observed in more diverse, low-density plantings, but the response magnitude varied among species. NRE increased as diversity increased; the response, however, varied among species, ranging from a statistically significant increase to no response, but was not influenced by planting density. These findings provide evidence that while the influence of diversity on tree physiological responses depends on the type of competitive interaction (i.e., diversity or intensity), different combinations of species will likely affect processes related with niche complementarity and, ultimately, a positive biodiversity–productivity relationship.

**Supplementary Information:**

The online version contains supplementary material available at 10.1007/s00442-026-05873-x.

## Introduction

Forest ecosystems play critical roles in many global processes, contributing approximately 50% of terrestrial net primary productivity and carbon storage (Bonan 2008; Mitchard 2018). Tree diversity in forests is declining globally and is expected to continue to decrease in the future due to increased rates of habitat fragmentation and land-use and climate change (Sax and Gaines 2003). Continued loss of biodiversity may lead to negative economic and environmental consequences, specifically related with ecosystem services, including nutrient cycling, nitrogen-use efficiency, and productivity (Brooks et al. 2002; Chapin et al. 2000; McCallum 2015). Declines in species diversity can influence multiple factors of ecosystem functioning that can impact water quality, soil health, and atmospheric carbon sequestration, and overall forest health (Bonan 2008; Humbert and Dorigo 2005; Jactel et al. 2018; Smith 2007; Tilman 1996). Understanding responses of individual trees to different levels of diversity and competition can help predict how the loss of biodiversity will influence forest population dynamics and community structure, and ultimately ecosystem functioning.

The influence of biodiversity on productivity, primarily measured as biomass accumulation, economic return, or carbon sequestration (Reich et al. 1997a, b; Tilman et al. 2001; Keltry 2006), is generally positive; this has been demonstrated across many terrestrial and aquatic systems (Aarseen 1997; Hector et al. 1999; Humbert and Dorigo 2005; Liang et al. 2015, 2016; Loreau 2000; Smith 2007; Spehn et al. 2000; Tilman and Downing 1994; Tilman 1996). The positive relationship between biodiversity and productivity has been estimated using both theory and mathematical modeling and quantified using experimental approaches (Loreau 1998; McCann 2000; Naeem et al. 1994). Experimental approaches, which use replicate plots with controlled levels of species diversity, have proven useful to identify biodiversity–productivity relationships (Tilman 1996; Huang et al. 2018; Isbell et al. 2009; Liang et al. 2016; Niklaus et al. 2017; Mason and Connolly 2020). Although there is considerable evidence supporting a positive biodiversity–productivity relationship, physiological mechanisms underpinning these relationships are not well understood.

Possible mechanisms driving biodiversity–productivity relationships have been debated for decades and range from complementarity (Loreau 2000) and facilitation effects to random chance based on selection effects (van Ruijven and Berendse 2005). Many studies have focused on the effects of species richness and evenness on resource dynamics, from which the concept of niche-use efficiency originated (Grossman et al. 2017; Liang et al. 2015, 2016; Naeem et al. 1994; Niklaus et al. 2017; Turnbull et al. 2013). Conceptually, niche-use efficiency is thought to be driven by species competition and performance across mono and polycultures and how these interactions are affected by light interception, precipitation patterns, nutrient availability, soil quality, and other abiotic factors that affect the uptake and use of resources (Liang et al. 2015). Inherently, niche-use efficiency is believed to be driven by a release of intraspecific competition and a more complementary use of resources as communities sharing resources become more diverse (Hodapp et al. 2019; Wang et al. 2005; Craine and Dybzinski 2013). An important limiting resource in closed-canopy forest systems is nutrient availability (Fernández-Martínez et al. 2014), and competition can influence nutrient dynamics in forest systems. Individuals experiencing interspecific competition have shown to more efficiently use available resources compared with individuals experiencing intraspecific competition in forests (Hooper and Vitousek 1998; Svanbäck and Bolnick 2007). While niche-use efficiency is thought to drive a positive biodiversity–productivity relationship, the physiological processes contributing to this relationship, especially ones related with nutrient uptake, are not well characterized.

A key component of nutrient dynamics that contributes to niche-use efficiency, especially in perennial systems, is the resorption and storage of nutrients before dormant periods. Perennial plants can resorb more than half of their existing foliar nitrogen prior to senescence (Vergutz et al. 2012), which can be reused for growth in the following season. By resorbing nitrogen, plants are less dependent on soil nutrient availability, a resource that can be under intense competition from neighboring plants and other species, especially in systems where nutrient inputs are limited or not available (Borer et al. 2015; Fay et al. 2015). In shrub and herbaceous systems, species richness influences nutrient resorption rates, where higher levels of species richness cause plants to more efficiently use foliar nutrients to produce biomass (Lü et al. 2019). However, how nutrient resorption, as a key mechanism of nutrient use efficiency, responds in longer-lived plant species (i.e., forest trees) under different levels of diversity and competition is not well understood.

In this study, we examined the influence of tree diversity and competition on nutrient dynamics in a hardwood plantation forest in a factorial design of mono and polycultures planted at different densities. We hypothesized that competition, both as species diversity (i.e., interspecific and intraspecific competition) and competition intensity (i.e., planting density) will influence nitrogen resorption efficiencies (NRE) of individual trees. We predicted that increased competition, either inter or intraspecific, implemented as closer planting densities, will lead to reduced canopy nitrogen levels, lower foliar litter nitrogen levels, and higher NRE of individuals, but these responses will depend on diversity. Specifically, we anticipate greater NRE values in trees in lower diversity plantings due to increased intraspecific competition for resource competition. We anticipate that outcomes from this work will advance our understanding of specific mechanisms that drive niche-use efficiency and help to better understand processes driving biodiversity–productivity relationships in forest systems.

## Methods

### Experimental design

This work was conducted in a mixed-species forest plantation at the Martell Forest research station (40.4°N, − 87.0°W) near Purdue University, West Lafayette, IN, USA. The plantation used in this study was established in 2007 and the site was previously used as an agricultural field. The site is moderately well-drained and productive, with the main soil type classified as Rockfield silt loam, with mean annual precipitation of ~ 1000 mm and mean annual temperature of ~ 10 °C (National Centers for Environmental Information 2025). The site has been managed consistently since plantation establishment, following standard forest management practices for weeding and deer management. The site includes three blocks, in which species combination plots were randomized, in a full-factorial design. Tree diversity was manipulated by varying the number of species (black cherry, *Prunus serotin*a; American chestnut, *Castanea dentata*; and northern red oak, *Quercus rubra*), and competition intensity was proxied by planting density, with trees planted at three different densities: 1 × 1 m spacing (10,000 trees ha^−1^), 2 × 2 m spacing (2500 trees ha^−1^), and 3 × 3 m spacing (1111 trees ha^−1^). Plot sizes for each planting density were 20 m^2^ for the 1 × 1 m spacing, 80 m^2^ for the 2 × 2 m spacing, and 180 m^2^ for the 3 × 3 m spacing. Within each block there are three single-species plots, three two-species plots, and one three-species plot per planting density. Because of substantial tree mortality in the 3 × 3 m spacing plots in one of the replicate blocks of the field site, we excluded this density treatment from field collections and analyses in the current study. Thus, with two densities, seven species combinations, and three replicate blocks the total number of plots within each block was 14 and the total number of plots used at the site was 42. Each plot within each block consisted of 56 trees, 26 of which were used as border row trees to separate plots and minimize edge effects. No sampling was conducted in border trees. Further details of the methods used for study establishment, site description, and experimental design can be found in Gauthier et al. (2013).

### Canopy foliar collections

In 2017, canopy foliar samples were collected at the end of midseason (late August—early September) using pole pruners. Within all plots for each block, three trees of each species within the plot were sampled. Leaves were collected from the upper and lower thirds of canopies of individual trees. Seven leaves from each canopy position were sampled from northern red oak and American chestnut trees; to collect a comparable mass of leaf tissue, twenty leaves were sampled per canopy position in black cherry trees. Thus, the total number of trees sampled from each plot varied depending on species combinations. In single-species plots three trees were sampled; in two-species plots six trees (2 species × 3 from each species = 6 trees) were sampled; and in three-species plots nine trees (3 species × 3 from each species = 9 trees) were sampled. The total number of foliar samples collected was 432 ((([6 single-species plots per block × 3 trees per plot] + [6 two-species plots per block × 6 trees per plot] + [2 three-species plots per block × 9 trees per plot]) × 2 canopy position) × 3 blocks = 432). After foliar collections, samples were flash frozen in liquid nitrogen then oven dried at 60° C until they reached a constant mass. Dried samples were ball milled and then processed for chemical analyses.

### Litter foliar collections

In 2017, at the start of leaf abscission (mid-September), leaf litter was collected weekly through full leaf drop (mid-November). Litter was collected using buckets with a diameter of 0.29 m and a collection area of 0.066 m^2^. Because of the different plot sizes, the number of buckets used was adjusted to represent a similar collection area in each plot: one bucket in the 10,000 trees ha^−1^ planting densities and four buckets in the 2500 trees ha^−1^ planting densities. After litter collections, leaves were sorted based on species and planting diversity and further sorted into three, equally spaced collection dates (September 11–29, October 01–20, and October 22-November 10). Litter samples were ball milled and then processed for chemical analyses.

### Foliar chemical analysis

Following foliar tissue and litter tissue collections described above, samples were analyzed for foliar nitrogen. Briefly, 28–32 mg of foliar and litter tissue was weighed into an aluminum tin and nitrogen was using combustion analysis using a Thermo Finnigan Flash 1112 Elemental Analyzer (San Jose, CA, USA). Atropine was used as a standard.

### Statistical analyses

Midseason foliar nitrogen concentrations were analyzed using a four-way analysis of variance (ANOVA) following the model y_*ij*_ = µ + b_*e*_ + B_*i*_ + S_*k*_ + D_*j*_ + P_*l*_ + BS_*ik*_ + BD_*ij*_ + BP_*il*_ + SD_*kj*_ + SP_*kl*_ + DP_*jl*_ + BSD_*ikj*_ + BDP_*ijl*_ + BSP_*ikl*_ + SDP_*kjl*_ + BSDP_*ikjl*_ + e_*ikjl*_. In this model, b represents the random effect of block *e*, B represents planting diversity *i*, S represents tree species *k*, D represents planting density *j*, P represents canopy position *l*, and *e* represents the error term. Litter nitrogen concentrations were analyzed using an ANOVA similar as the one used for analysis of midseason nitrogen concentrations, except that we substituted time (*t*_*x*_) for canopy position as a fixed effect.

Nitrogen resorption efficiency (NRE) was calculated using the following equation following Vergutz et al. 2012:

$$NRE \left(\%\right)=\left(\frac{Nms-Nl}{Nms}\right) * 100.$$

In this equation, *Nms* is the nitrogen concentration of leaf tissue at midseason and *Nl* is the nitrogen concentration of the litter. Midseason foliar nitrogen was averaged over canopy positions to use the tree as the biological replicate. NRE was analyzed using a similar ANOVA as the one used for the analysis of litter nitrogen concentrations, with the exception that we excluded litter sampling period as a fixed effect in the final analysis of NRE, because the main effect of sampling period and all interactions were not statistically significant in the analysis of NRE. A Tukey post hoc test was performed for comparisons with significant terms in the ANOVA model. Relationships between midseason foliar nitrogen and litter nitrogen concentrations with NRE were assessed using an analysis of covariance (ANCOVA), with species as a factor and either midseason foliar nitrogen or litter nitrogen as a covariate, midseason foliar nitrogen data averaged across canopy levels, and litter data averaged over sampling period for the same reason as above. We acknowledge that both midseason foliar nitrogen and litter nitrogen values were used in the calculation of NRE, potentially introducing correlation among variables. Because NRE is mechanistically derived from these component variables, however, excluding them would preclude evaluating species-level differences in nutrient resorption strategies. Moreover, examining these relationships using a consistent analytical framework across species allows for meaningful comparisons, even when component variables are mathematically related. Our approach is an effort to emphasize relative patterns among species, rather than independence among predictors, and provides necessary ecological context for interpreting interspecific variation in nitrogen-use strategies.

Examination of residuals confirmed that midseason and litter nitrogen concentrations and NRE values followed normal distributions. To balance between type I and II errors, we considered *P* values < 0.05 as significant and 0.05 ≤ *P* ≤ 0.10 as marginally significant. All statistical analyses were performed using JMP v.19 (SAS Institute, Cary, NY 2018).

## Results

### Midseason foliar nitrogen levels

**Fig. 1:**
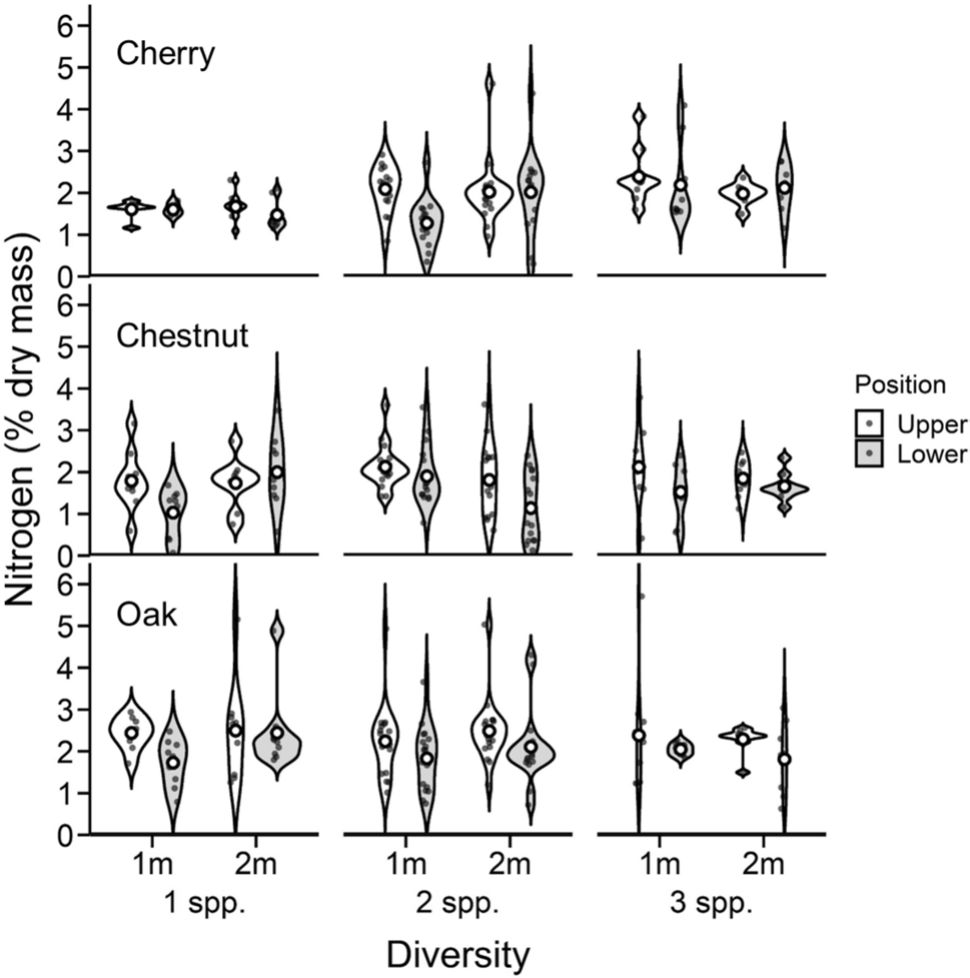
Influence of diversity, planting density, and canopy position on midseason (late August–early September) foliar nitrogen concentrations across species. Violin plots represent the distribution of nitrogen concentrations across all possible combinations of diversity (one-, two-, and three-species combinations; 1 spp., 2 spp., and 3 spp., respectively), planting density (1- and 2-meter spacing; 1 m and 2 m, respectively), canopy layer (upper third of canopy, white violins; lower third of canopy, gray violins), and tree species (cherry, top panel; chestnut, middle panel; oak, bottom panel). Broadness of each violin indicates data density and distribution, and open circles within violins represent mean values

Overall, canopy nitrogen concentrations varied among tree species, but nitrogen levels within species were influenced by diversity levels, planting density, and canopy positions (Table 1; SI Table 1). Overall, oak trees had ~ 20% more foliar nitrogen than cherry or chestnut trees and foliar nitrogen was ~ 17% greater in the upper, compared with lower, canopies across all tree species (SI Table 1; Fig. 1). The influence of diversity on foliar nitrogen levels varied among species and planting density (Table 1; Fig. 1). Cherry and chestnut trees had greater foliar nitrogen levels as diversity increased, while northern red oaks trees had little response to diversity levels (SI Table 1; Fig. 1). Foliar nitrogen levels tended to increase in the more spacious planting density, but the response varied among species and diversity levels, with cherry generally increasing, and oak decreasing, nitrogen levels as density increased (Table 1; Fig. 1). Our results showed a marginally significant interaction between diversity, species, planting density, and canopy position, suggesting that the influence of diversity and density on canopy nitrogen levels was larger in leaves from the upper, compared with lower, canopies, but the magnitude of this response was more pronounced in cherry and chestnut, and less pronounced in northern red oak, and more so in the larger planting density (Table 1; Fig. 1).

**Table 1:** Summary of F and *P* values for influence of diversity, species identity, planting density, and canopy position on midseason foliar nitrogen concentrations using a full-factorial ANOVA with block as a random effect. Df, degrees of freedom (numerator, denominator)

Treatments and interactions	*Df*	F	*P*
Diversity	2, 374	2.02	0.134
Species	2, 374	14.52	< 0.001
Diversity × species	4, 374	2.22	0.062
Density	1, 374	0.33	0.565
Diversity × density	2, 374	2.44	0.088
Species × density	2, 374	0.78	0.455
Diversity × species × density	4, 374	3.39	0.009
Canopy position	1, 374	17.68	< 0.001
Diversity × canopy position	2, 374	0.17	0.492
Species × canopy position	2, 374	0.78	0.486
Diversity × species × canopy position	4, 374	0.28	0.887
Density × canopy position	1, 374	3.09	0.079
Diversity × density × canopy position	2, 374	0.61	0.542
Species × density × canopy position	2, 374	0.08	0.920
Diversity × species × density × canopy position	4, 374	2.40	0.049

*Df* degrees of freedom (numerator, denominator)

*P* values < 0.05 are bold and *P* values 0.05 < *P* < 0.10 are italicized

### Litter nitrogen levels

**Fig. 2:**
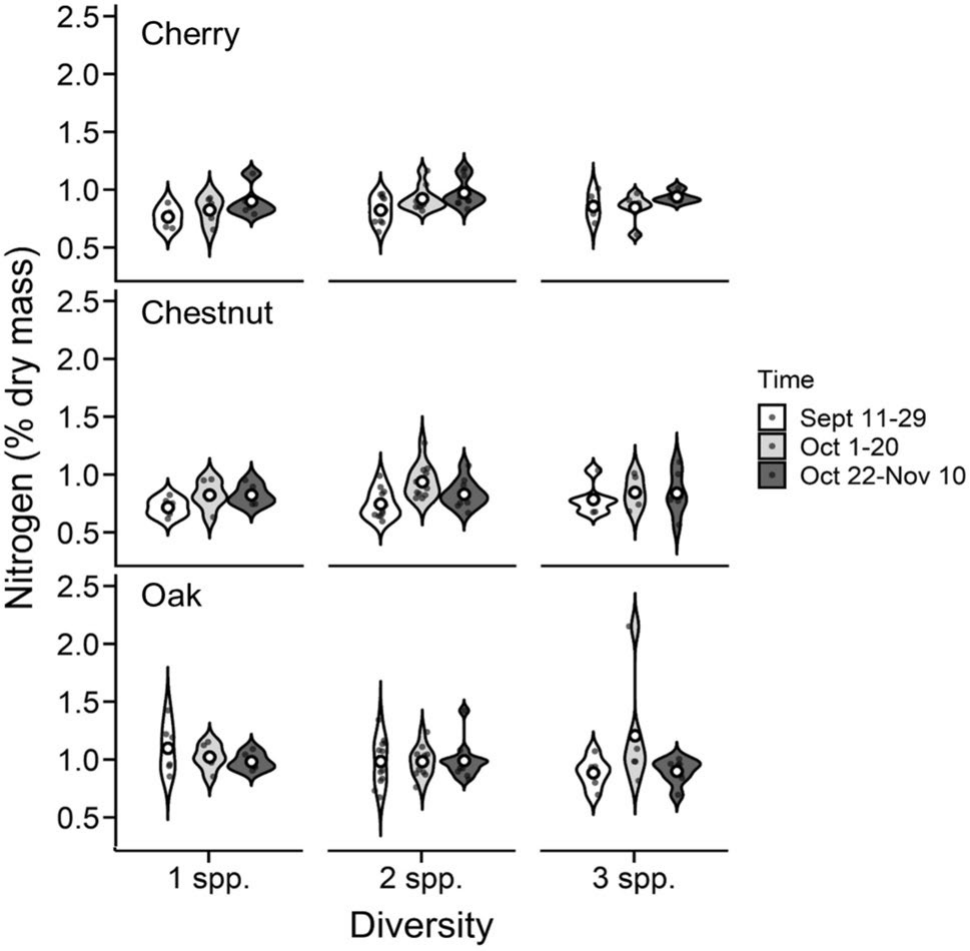
Influence of diversity and collection period on foliar litter nitrogen concentrations across species. Violin plots represent the distribution of nitrogen concentrations across all possible combinations of diversity (one-, two-, and three-species combinations; 1 spp., 2 spp., and 3 spp., respectively), collection period (Sept 11–29, white violins; Oct 1–20, gray violins; Oct 22-Nov 10, black violins), and tree species (cherry, top panel; chestnut, middle panel; oak, bottom panel). Broadness of each violin indicates data density and distribution, and open circles within violins represent mean values

Foliar litter nitrogen levels varied among tree species and collection periods (Table 2; SI Table 2; Fig. 2). Litter nitrogen levels were generally higher in oak and more consistent over the collection periods, compared with cherry and chestnut, where lower levels of litter nitrogen occurred in the earlier, compared with later, collection periods (Fig. 2). A marginally significant interaction of diversity and species was revealed (Table 2), suggesting that the variation among tree species depended on the diversity level; oak and chestnut litter maintained constant nitrogen levels over the different diversity levels, while cherry trended to increase nitrogen concentrations in litter as diversity levels increased, and more so in later collection periods (significant diversity × species × collection period interaction; Fig. 2, SI Table 2).

**Table 2:** Summary of F and *P* values for foliar litter nitrogen concentrations using a full-factorial ANOVA with block as a random effect. Df, degrees of freedom (numerator, denominator)

Treatments and interactions	*Df*	F	*P*
Diversity	2, 152	1.79	0.169
Species	2, 152	24.52	< 0.001
Diversity × species	4, 152	2.26	0.068
Density	1, 152	0.01	0.936
Diversity × density	2, 152	0.46	0.626
Species × density	2, 152	0.12	0.882
Diversity × species × density	4, 152	0.77	0.546
Collection period	2, 152	4.38	0.014
Diversity × collection period	4, 152	0.71	0.582
Species × collection period	4, 152	1.26	0.288
Diversity × species × collection period	8, 152	2.01	0.048
Density × collection period	2, 152	0.37	0.686
Diversity × density × collection period	4, 152	0.35	0.840
Species × density × collection period	4, 152	0.37	0.826
Diversity × species × density × collection period	8, 152	1.07	0.380

*Df* degrees of freedom (numerator, denominator)

*P* values < 0.05 are bold and *P* values 0.05 < *P* < 0.10 are italicized

### Nitrogen resorption efficiency

**Fig. 3:**
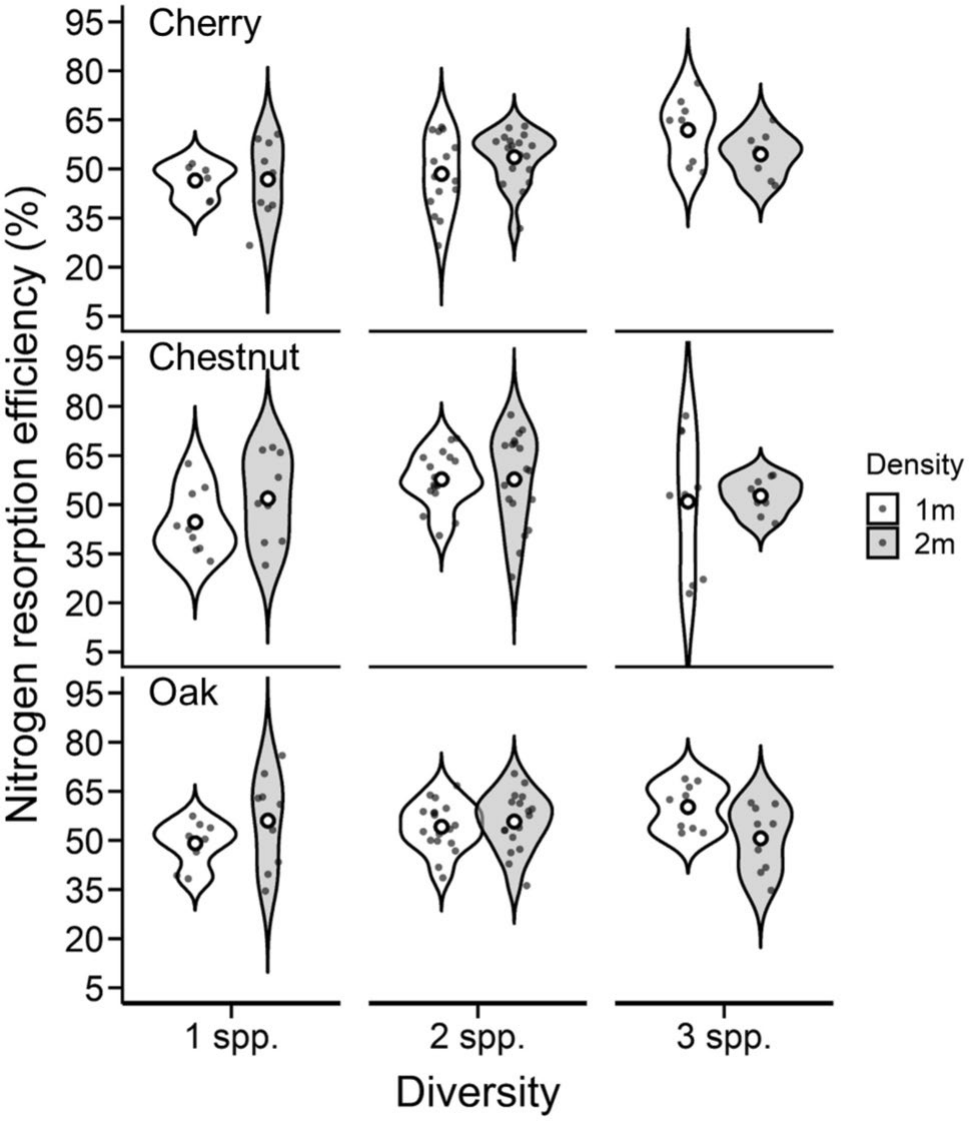
Influence of diversity and planting density on nitrogen resorption efficiencies across species. Violin plots represent the distribution of nitrogen resorption efficiency across all possible combinations of diversity (one-, two-, and three-species combinations; 1 spp., 2 spp., and 3 spp., respectively), planting density (1- and 2-meter spacing; 1 m and 2 m, white and gray violins, respectively), and tree species (cherry, top panel; chestnut, middle panel; oak, right panel). Broadness of each violin indicates data density and distribution, and open circles within violins represent mean values

NRE was influenced by diversity level, with NRE values increasing as diversity increased (Table 3; SI Table 3; Fig. 3). We found no difference between the two- and three-species plantings, but on average, NRE increased by ~ 12% when comparing NRE in the single-species planting with NRE in the three-species plantings (Table 3; SI Table 3; Fig. 3). The magnitude of response of NRE across diversity levels, however, varied among tree species (significant diversity × species interaction Table 3), with black cherry having the strongest release from intraspecific competition (23% increase from single- to three-species planting, Fig. 3). While both American chestnut (14% increase from single- to three-species planting) and northern red oak (8% increase from single- to three-species planting) increased NRE in more diverse settings, the results were not statistically significant (Fig. 3). We did not find an influence of density, or any interactions with other factors, on NRE, suggesting that more intense competition (i.e., closer planted individuals) did not affect the type of competition (i.e., intra- or interspecific competition).

**Table 3:** Summary of *F* and *P* values for nitrogen resorption efficiencies using a full-factorial ANOVA with block as a random effect. Df, degrees of freedom (numerator, denominator)

Treatments and interactions	*Df*	F	*P*
Diversity	2, 51	5.57	0.006
Species	2, 51	0.30	0.738
Diversity × species	4, 51	2.70	0.040
Density	1, 51	0.28	0.598
Diversity × density	2, 51	0.91	0.410
Species × density	2, 51	0.14	0.870
Diversity × species × density	4, 51	0.73	0.572

*Df* degrees of freedom (numerator, denominator)

*P* values < 0.05 are bold and *P* values 0.05 < *P* < 0.10 are italicized

### Relationships of midseason and litter nitrogen with NRE across species

**Fig. 4:**
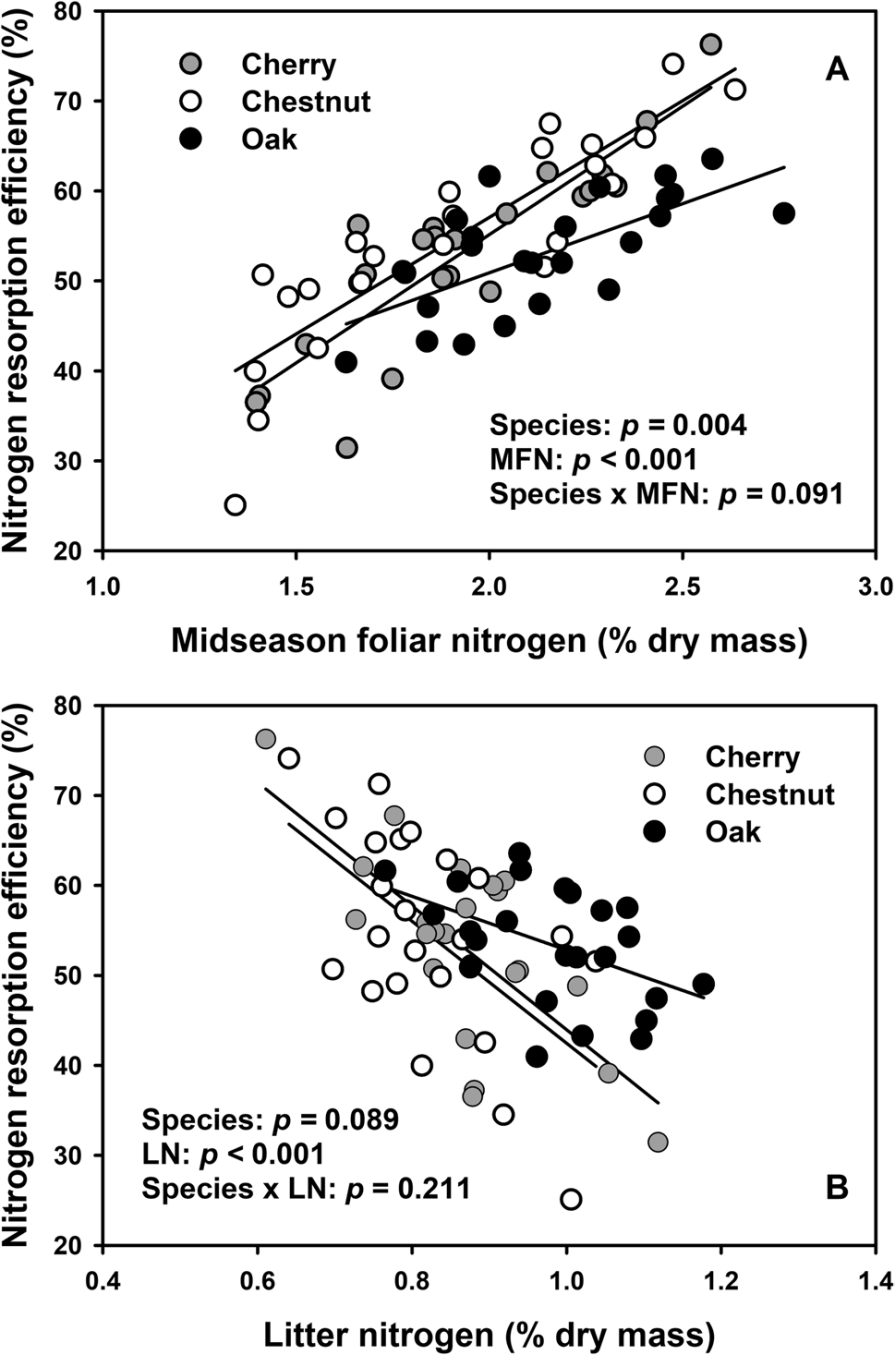
Relationships between (**a**) midseason foliar nitrogen levels and nitrogen resorption efficiencies (NRE) and (**b**) litter nitrogen levels and NRE for cherry (open circles), chestnut (gray circles), and oak (black circles). *MFN* midseason foliar nitrogen, *LN* litter nitrogen

Midseason foliar nitrogen concentrations were positively related with NRE and the response varied across tree species (Fig. 4a). The relationship between midseason foliar nitrogen and NRE was less pronounced for northern red oak than for black cherry and chestnut (marginally significant species × midseason foliar nitrogen concentration interaction, Fig. 4a). Litter nitrogen concentrations varied among tree species and were negatively related with NRE values, but the response was consistent across tree species (Fig. 4b).

## Discussion

An increase in productivity as plant diversity increases is thought to be sustained by resource complementarity, or niche-use efficiency, by individuals in a community (Liang et al 2015). As biodiversity increases, individuals within a community can more efficiently use available nutrients, water, and light than if conspecifics, which use the resources more similarly, exist in the same space (Grossiord 2018; Hodapp et al. 2019). This predicted release of competition with increasing diversity could expand available niche space to accommodate a more diverse set of species. While niche-use efficiency has been promoted as a driver of the biodiversity–productivity relationship (Liang et al. 2015), specific mechanisms underpinning niche-use efficiency are not well characterized. Here, we show that a release from intraspecific competition (i.e., increased species diversity and lower planting density) increased the efficiency of nitrogen resorption in individual trees, although the response varied among different tree species. While these results indicate a potential physiological mechanism for niche complementarity promoting a positive biodiversity–productivity relationship, they highlight that not all combinations of tree species will respond similarly with implications for a generalized positive biodiversity–productivity response.

In agreement with our predictions, our results suggest that the influence of diversity on midseason foliar nitrogen levels varied among tree species and planting densities. Black cherry trees increased, while chestnut and oak trees showed little increase to no changes, respectively, in midseason foliar nitrogen levels as planting densities increased, and the response from cherry was greater in more species-rich environments. These findings suggest that while intraspecific competition can have an influence on nutrient uptake during the growing season, the impact can be more pronounced for some tree species than others, regardless of the intensity of the intra and interspecific competition, and that individual tree species or different combinations of tree species might respond differently to release of competition (Postma et al. 2020; Rehil et al. 2020). We also found that midseason foliar nitrogen concentrations were positively correlated with NRE values, and the response varied among tree species. Higher midseason foliar nitrogen concentrations have been shown to be positively correlated with higher nitrogen resorption efficiency rates, an outcome suggested to be due to higher nitrogen levels in leaves providing a greater pool of nitrogen for remobilization before senescence (Aerts 1997; Killingbeck 1996). While a caveat of this work is the limited time series of data collected, such species-specific responses have significant implications for how different tree species assemblages potentially influence positive biodiversity–productivity relationships (Adler et al. 2018; Mahaut et al. 2020; Zhang et al. 2024).

In partial agreement with our predictions, the influence of species richness on litter nitrogen concentrations differed among species, with black cherry showing a statistically significant increase, while changes in the other species were not statistically significant. The range in the increase from monocultures to three-species plantings across species was 6–10%. In general, lower litter nitrogen concentrations can suggest more efficient resorption of nutrients prior to leaf senescence and higher litter nitrogen levels can accelerate litter decomposition and nitrogen mineralization (Aerts 1997; Aerts and Chapin 2000). We also found a negative relationship between litter nitrogen levels and NRE values. A negative relationship between foliar litter nitrogen content and NRE is likely due to more efficient nitrogen resorption resulting in lower nitrogen remaining in senescing leaves (Killingbeck 1996; Vergutz et al. 2012). This pattern could be an indicator of internal nutrient recycling, where plants with high NRE withdraw more nitrogen before leaf drop, leaving behind litter with reduced nutrient content. Considering two potential nutrient cycling pathways, leaf nitrogen resorption and litter nitrogen mineralization, leaf nitrogen resorption is suggested to potentially be more influential than litter nitrogen mineralization in maintaining a positive diversity–productivity relationship in nutrient limited environments (Zhang et al. 2023).

Our findings contrast with those of others, where species richness had a negative relationship with green leaf and litter nitrogen content (Lü et al. 2019). One possible reason for this contrast is that the maximum number of species included in the current study was limited to three, whereas other studies considering nutrient dynamics have included many more levels of diversity (Tillman et al. 2001; Fornara et al. 2009; Lü et al. 2019). When considering only the lower number of species included in the overall response across all levels of diversity in many of these studies, however, a positive diversity–productivity trend is typically small, disappears, or is negative (Tillman et al. 2001; Liang et al. 2015), suggesting that consideration of the biodiversity–productivity relationship is context dependent, either with the number of species included or the relatedness of niche space of the species considered.

In contrast with our predictions, our findings indicate that as plant diversity increased, NRE also increased. Like the other findings of this study, however, the response magnitude again varied among tree species. While an increase in NRE as diversity increases is consistent with findings by Lü et al. (2019), who found an increase in NRE as diversity increased in a grassland experimental system, the species-specific nature of the outcomes found in this study suggest not all combinations of tree species will respond similarly. This outcome might potentially be because of the lack of niche similarity, with implications for limiting a positive diversity–productivity relationship. Indeed, analysis of over a decade of growth at this plantation revealed no positive relationship between diversity and productivity (Blackstone et al. 2025). The concept of niche overlap, or species niche complementarity, among different species has the potential to generate differential capacities for species to coexist and will likely influence a consistent outcome of a positive biodiversity–productivity relationship (Liang et al. 2015; Blackstone et al. 2025). While no variation in nutrient availability was found across this site (Blackstone et al. 2025), including other factors, such as photosynthetic and water-use parameters, and available soil nitrogen, studies examining biodiversity–productivity relationships will be useful in discovering similarities and differences in mechanisms driving changes in the relationships between diversity and productivity across systems. This work highlights that the influence of complementarity to the contribution of niche-use efficiency, especially via nutrient dynamics, on biodiversity–productivity relationships can be species or system specific with potential consequences for forest productivity.

## Supplementary Information

Below is the link to the electronic supplementary material.

Supplementary file1 (DOCX 21 KB)

## Data Availability

Data used in this manuscript are available online from the Purdue University Research Repository (PURR). Data set citation: Nelson TM, Park M, Cotrozzi L, Blackstone KMS, McNickle GG, Jacobs DF, Couture JJ (2025) Data for Species diversity and competition influence nitrogen resorption efficiency in mixed hardwood plantations. DOI: https://purr.purdue.edu/publications/5004/1.
