# Supplementary material for: Species diversity and competition influence nitrogen resorption efficiency in mixed hardwood plantations
Source: Oecologia. 2026 Mar 14;208(3):42. doi: 10.1007/s00442-026-05873-x (PMC12992424; doi:10.1007/s00442-026-05873-x)
Supplement: Supplementary file 1 — Supplementary file1 (DOCX 21 KB) [file 442_2026_5873_MOESM1_ESM.docx]

SI Table 1. Average midseason foliar nitrogen concentrations for black cherry (*Prunus serotina*), American chestnut (*Castanea dentata*), and northern red oak (*Quercus rubra*) in all combinations of diversity (one, two, and three species combinations) and planting density (one-meter spacing and two-meter spacing) used in this study. Values are mean ± one standard error.

|  |  |  |  |  |  |  |  |
| --- | --- | --- | --- | --- | --- | --- | --- |
|  |  | **Diversity** | | | | | |
|  |  | **One species** | | **Two species mixture** | | **Three species mixtures** | |
| **Species** | **Canopy Position** | **1m** | **2m** | **1m** | **2m** | **1m** | **2m** |
| **Cherry** | **Upper** | 1.6±0.3 | 1.7±0.2 | 2.1±0.2 | 2.0±0.2 | 2.4±0.2 | 2.0±0.3 |
|  | **Lower** | 1.6±0.3 | 1.5±0.2 | 1.3±0.2 | 1.9±0.2 | 2.2±0.2 | 2.1±0.3 |
| **Chestnut** | **Upper** | 1.8±0.2 | 1.7±0.2 | 2.1±0.2 | 1.8±0.2 | 2.1±0.2 | 1.8±0.2 |
|  | **Lower** | 1.0±0.2 | 2.0±0.2 | 1.9±0.2 | 1.1±0.2 | 1.5±0.2 | 1.7±0.2 |
| **Oak** | **Upper** | 2.4±0.2 | 2.5±0.2 | 2.2±0.2 | 2.5±0.2 | 2.4±0.2 | 2.3±0.2 |
|  | **Lower** | 1.7±0.2 | 2.4±0.2 | 1.8±0.2 | 2.1±0.2 | 2.1±0.2 | 1.8±0.2 |

SI Table 2. Average litter nitrogen concentrations (% DM) for black cherry black cherry (*Prunus serotina*), American chestnut (*Castanea dentata*), and northern red oak (*Quercus rubra*) in all combinations of diversity (one, two, and three species combinations) and planting density (one-meter spacing and two-meter spacing) used in this study collected at different time periods. Values are mean ± one standard error.

|  |  |  |  |  |  |  |  |
| --- | --- | --- | --- | --- | --- | --- | --- |
|  |  | **Diversity** | | | | | |
|  |  | **One species** | | **Two species mixture** | | **Three species mixtures** | |
| **Species** | **Collection Period** | **1m** | **2m** | **1m** | **2m** | **1m** | **2m** |
| **Cherry** | **11-29 September** | 0.8±0.1 | 0.7±0.1 | 0.8±0.1 | 0.8±0.1 | 0.8±0.1 | 0.9±0.1 |
|  | **01-20 October** | 1.0±0.1 | 0.8±0.1 | 1.0±0.1 | 0.9±0.1 | 0.8±0.1 | 0.9±0.1 |
|  | **22 October 10 November** | 0.9±0.1 | 0.9±0.1 | 1.0±0.1 | 1.0±0.1 | 0.9±0.2 | 1.0±0.1 |
| **Chestnut** | **11-29 September** | 0.7±0.1 | 0.8±0.1 | 0.8±0.1 | 0.7±0.1 | 0.8±0.1 | 0.7±0.1 |
|  | **01-20 October** | 0.8±0.1 | 0.9±0.1 | 1.0±0.1 | 1.0±0.1 | 0.8±0.1 | 0.9±0.1 |
|  | **22 October 10 November** | 0.8±0.1 | 0.8±0.1 | 0.8±0.1 | 0.9±0.1 | 0.9±0.1 | 0.8±0.1 |
| **Oak** | **11-29 September** | 1.2±0.1 | 1.0±0.1 | 1.0±0.1 | 1.0±0.1 | 0.8±0.1 | 1.0±0.1 |
|  | **01-20 October** | 1.0±0.1 | 1.0±0.1 | 1.0±0.1 | 1.0±0.1 | 1.3±0.1 | 1.1±0.1 |
|  | **22 October 10 November** | 1.0±0.1 | 1.0±0.1 | 1.0±0.1 | 1.0±0.1 | 0.8±0.1 | 0.9±0.1 |

SI Table 3: Average nitrogen resorption efficiencies (%) for black cherry (*Prunus serotina*), American chestnut (*Castanea dentata*), and northern red oak (*Quercus rubra*) in all combinations of diversity (one, two, and three species combinations) and planting density (one-meter spacing and two-meter spacing) used in this study collected at different time periods. Values are mean ± one standard error.

|  |  |  |  |  |  |  |
| --- | --- | --- | --- | --- | --- | --- |
|  | **Diversity** | | | | | |
|  | **One species** | | **Two species mixture** | | **Three species mixtures** | |
| **Species** | **1m** | **2m** | **1m** | **2m** | **1m** | **2m** |
| **Cherry** | 44.3±4.8 | 51.8±4.1 | 45.9±3.4 | 55.8±3.3 | 61.3±4.7 | 57.7±3.9 |
| **Chestnut** | 49.3±4.1 | 51.9±4.1 | 58.6±3.3 | 56.7±3.3 | 50.1±4.1 | 52.6±4.1 |
| **Oak** | 49.3±4.1 | 52.5±4.1 | 48.6±3.3 | 56.7±3.3 | 57.5±3.9 | 51.7±4.1 |
